# Inhibition of MMP2 activity mitigates *N*-omega-nitro-l-arginine-methyl ester (l-NAME)-induced right heart failure

**DOI:** 10.1016/j.redox.2024.103308

**Published:** 2024-08-15

**Authors:** Rolf Schreckenberg, Rainer Schulz, Nadja Itani, Peter Ferdinandy, Peter Bencsik, Tamara Szabados, Susanne Rohrbach, Bernd Niemann, Klaus-Dieter Schlüter

**Affiliations:** a Institute of Physiology, JLU Giessen, 35392, Giessen, Germany; b Department of Pharmacology and Pharmacotherapy, University of Szeged, Szeged, Hungary; c Universitätsklinikum Gießen, Klinik für Herz-, Kinderherz- und Gefäßchirurgie, 35392, Gießen, Germany

**Keywords:** Superoxide dismutase, Myocardial hypertrophy, Cardiac fibrosis

## Abstract

In rats decreased bioavailability of nitric oxide induces oxidative stress and right heart failure. Oxidative stress can activate matrix metalloproteinase-2 (MMP2). We addressed the question whether increasing oxidative defense by administration of the SOD mimetic Tempol or direct inhibition of MMP2 activity by SB-3CT mitigates right heart failure. Rats received l-NAME for four weeks and during week three and four treatment groups received either Tempol or SB-3CT in addition. After four weeks heart function was analyzed by echocardiography, organ weights and expression of *NPPB* and *COL1A1* were analyzed, oxidative stress was monitored by DHE-staining and MMP2 activity was quantified by proteolytic auto-activation, zymography, and troponin I degradation. l-NAME induced oxidative stress and MMP2 activity stronger in the right ventricle than in the left ventricle. Troponin I, a MMP2 substrate, was degraded in right ventricles. Tempol reduced oxidative stress and preferentially affected the expression of fibrotic genes (i.e. *COL1A1*) and fibrosis. Tempol and SB-3CT mitigated right but not left ventricular hypertrophy. Neither SB-3CT nor Tempol alone strongly improved right ventricular function. In conclusion, both MMP2 activity and oxidative stress contribute to right ventricular failure but neither is MMP2 activation linked to oxidative stress nor does oxidative stress and MMP2 activity have common targets.

## Highlights

• Adminstration of l-NAME for four weeks causes oxidative stress in the right ventricle whereas the left ventricle can tolearte this stress.
• The SOD mimetic Tempol is sufficient to reduce oxidative stress in the right ventricle and attenuates the induction of pro-fibrotic pathways.
• The MMP2-inhibitor SB-3CT, however, does not affect oxidative stress but improves l-NAME-dependent maladaptation in cardiomyocytes.
• Improving oxidative defense of the RV affects fibrosis whereas MMP2 activation affects cardiomyocytes.

## Graphical abstract

**Figure:**
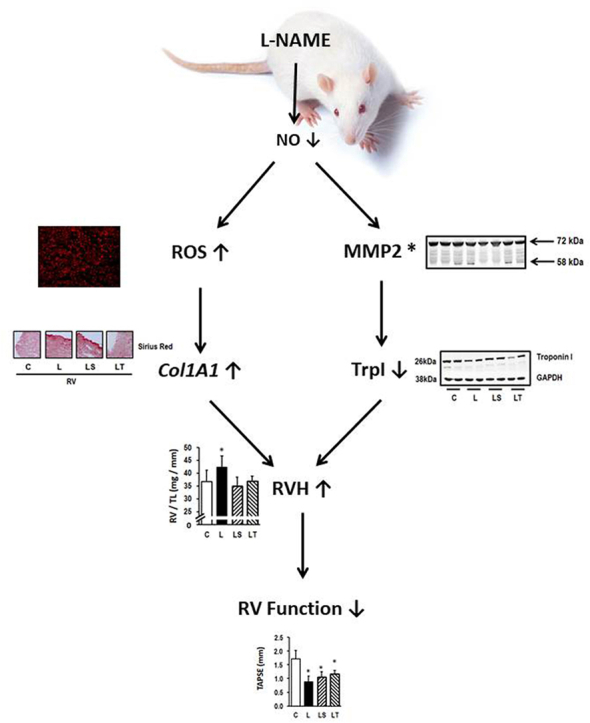

## 1 Introduction

Pressure overload of the left ventricle (LV) leads to cardiac hypertrophy that is initially compensatory. Therefore, pressure overload to the LV, as it occurs in primary hypertension, can be tolerated for years before it finally leads to heart failure. In contrast to LV the right ventricle (RV) of adults cannot compensate pressure overload for long but decompensates and develops right heart failure [[1], [2], [3]]. From these findings it is concluded that the underlying molecular adaptations to pressure load differ between both ventricles. This may be linked to their distinct embryological origin, to physiological differences (RV is designed for high volume low pressure work whereas the LV is designed to induce high pressure), to an un-proportional relative increase of pressure overload in the RV in pulmonary arterial hypertension (PAH) patients in comparison to pressure load increase of the LV in patients with systemic hypertension, or a different signaling in both ventricles in response to pressure overload [[4], [5], [6], [7]]. A better understanding about the adaptation of the right ventricle to pressure load is required to develop therapeutic options for such patients.

We established a rat model in which the LV of the heart develops a compensatory type of hypertrophy whereas the RV develops right heart failure in parallel [8]. The model is based on the oral application of *N*-omega-nitro-l-arginine-methyl ester (l-NAME), a nitric oxide synthase (NOS) inhibitor that induces systemic hypertension within two weeks [8]. Within four weeks all rats develop LV hypertrophy with preserved function whereas the RV exhibits signs of decompensation. l-NAME is added to the tap-water of the rats, thereby offering a minimal invasive model of right heart failure. On the molecular level we found previously that the LV ameliorates oxidative stress induced by l-NAME via upregulation of superoxide dismutase (SOD) whereas the RV was unable to suppress oxidative stress caused by l-NAME [8]. This makes the model to an ideal tool for investigation of molecular differences between the RV and LV in stress response.

To improve our understanding of l-NAME-induced right heart failure we extended our former study and addressed now two questions. First, does administration of SOD mimetics, such as 4-Hydroxy-TEMPOL (Tempol), mitigate right heart failure in this model? Second, can we identify MMP2 as a downstream target of oxidative stress in the RV? Our interest to understand the relationship between ROS and MMP2 activity in right heart failure is based on the following previous findings: Reactive oxidative species (ROS) activate MMP2 linking oxidative stress to MMP2 activity [9]. Several reports in the literature suggest a contribution of MMP2 to right heart failure [[10], [11], [12], [13]]. Furthermore, MMP2 targets troponin I, a sarcomere protein, indicating that MMP2 does not only modify extracellular matrix composition but also target cardiomyocytes directly [14,15]. Finally, MMP2 directly contributes to the regulation of heart weight in vitro [16]. Based on these findings, MMP2 seems to be an excellent candidate that participates in the transition of oxidative stress into right heart failure [17]. Here we used the MMP2 inhibitor SB-3CT to study the role of MMP2 in this model [18].

## 2 Materials and methods

### 2.1 Animal model

The investigation conforms to the Guide for the Care and Use of Laboratory Animals published by the US National Institute of Health (NIH Publication 85–23, revised 1996). The study was approved by the local authorities for animal experiments (V 54–19 c 20 15 h 01 GI 20/1 Nr. G 60/2018).

Forty-two twelve-weeks-old female Wistar rats were divided into four groups: Controls (C; drinking-water only; n = 12), l-NAME (L; 7.5 mg/day in drinking water), l-NAME plus SB-3CT (LS; SB-3CT during day 15–28; intraperitoneal injection every second day (25 mg/kg), and l-NAME plus Tempol (1.0 mM/L in drinking water during day 15–28). Rats were acclimatized to the housing facility two weeks prior to the onset of the experiments. All rats were euthanized at day twenty-eight by cervical dislocation under deep anesthesia (isoflurane).

The health status of the experimental animals was determined daily, using a ‘distress score’ (see ref. 8). Over the entire experimental period, no animals died or had to be excluded from the study based on the exclusion criteria. The inclusion criteria were an age of twelve weeks and a female sex. The rats were randomized into the four groups. Predefined exclusion criteria are a Distress-Score above 5 that did not appear in any of the rats used in this study.

All subsequently described procedures are established in the laboratory and only slightly modified for adjustment to the new samples.

### 2.2 Determination of blood pressure and heart size

Peak systolic blood pressure, diastolic blood pressure, and heart rate were measured weekly using the non-invasive tail-cuff method (TSE-Systems. 209000 Series). Before the start of the blood pressure measurements, the animals were adjusted to the experimental procedure over one week. The mean of 10 consecutive blood pressure readings was obtained for each animal. As blood pressure measurements were performed before with the same model and method [8,19] only a couple of rats were randomly selected to investigate again the effect of l-NAME administration to blood pressure (C, n = 3; L, n = 6; LS, n = 3; LT, n = 4). Results are given for systolic blood pressure and diastolic blood pressure.

### 2.3 Functional characterization by echocardiography

For echocardiographic analysis, rats were anaesthetized by isoflurane inhalation (2 %; 98 % O_2_). Two-dimensional and M-mode echocardiographic examinations were performed in accordance with the criteria of the American Society of Echocardiography using Vevo 2100 high-frequency high-resolution ultrasound system with a 13–24 MHz transducer probe (FUJIFILM VisualSonics Inc., Toronto, Canada), evaluating both cardiac geometry and function. Data are presented as right ventricular end-diastolic area (RVED Area), right ventricular end-systolic area (RVES Area), RV percent area change, and tricuspid annular plane systolic excursion (TAPSE).

### 2.4 MMP2 activity (zymography)

Gelatinolytic activity of MMP was examined on 8 % polyacrylaminde gels, co-polymerized with gelatin (2 mg/ml). Samples were loaded with 50 μg of protein. After electrophoresis, gels were washed with renaturation buffer (containing 2.5 % Triton X-100), and then incubated in development buffer to eliminate Triton X-100. Gels were stained with 0.05 % Coomassie Brilliant Blue, and gelatinolytic activities were detected as transparent bands against the dark background. Band intensities were quantified (Quantity One Software, BioRad, Hercules, CA) and expressed in arbitrary units.

### 2.5 Western blot

Total protein was extracted from right and left ventricles using Cell Lysis Buffer (10*x*) (Cell Signaling) according to the manufacturer's protocol. The homogenate was centrifuged at 14,000 g at 4 °C for 10 min and the supernatant was treated with Laemmli buffer. Protein samples were loaded on NuPAGE Bis-Tris Precast gels (10 %, Life Technology) and subsequently transferred onto nitrocellulose membranes. Blots were incubated with the following antibodies: MMP2 polyclonal antibody (Bioss Antibodies; bs-0412 R) and troponin I polyclonal antibody (Invitrogen; AB_2,546,440). Secondary antibody (horseradish peroxidase (HRP)-conjugated) directed against rabbit IgG was purchased from Dako (Agilent Technologies, Inc.). The immunoblot band intensities were quantified using Chemi-Capt 5000 and expressed as the molecule/GAPDH ratio.

### 2.6 RNA isolation and RT-PCR analysis

Total RNA was isolated from the RV and LV using peqGoldTriFast (peqlab; Biotechnologie GmbH) according to the manufacturer's protocol. To remove genomic DNA contamination, isolated RNA samples were treated with 1 U DNase/μg RNA (Invitrogen) for 15 min at 37 °C. One μg of total RNA was used in 10 μl reaction buffer to synthesize cDNA using Supercript RNaseH Reverse Transcriptase (200 U/μg RNA; Invitrogen) and oligo dTs as primers. RT reactions were performed for 50 min at 37 °C. Real-time quantitative PCR was performed using the MyiQ® detection system (Bio-Rad) in combination with the iTaq Universal SYBR Green Real-Time PCR Supermix (Bio-Rad). Quantification was performed as described by ref. [20] and expressed as x-fold of the median expression of the respective controls. Primer sequences are listed in Table 1.

**Table 1:** List of primers used in this study.

Gene	Forward	Reverse	Identification Number
B2M	GCCGTCGTGCTTGCCATTC	CTGAGGTGGGTGGAACTGAGAC	NM_012512.2
NPPB	ATGATTCTGCTCCTGCTTTTCC	TCTGCATCGTGGATTGTTCTG	NM_031,545
Col1A1	GCGAACAAGGTGACAGAG	CCAGGAGAACCAGCAGAG	NM_053,304
MMP2	ACAACAGCTGTACCACCGAG	GGACATAGCAGTCTCTGGGC	NM_031054.2
MMP12	TGCAGCTGTCTTTGATCCAC	GCATCAATTTTTGGCCTGAT	NM_053,963
UCP2	AGCAGTTCTACACCAAGGGC	GTGCTCTGGTATCTCCGACC	NM_19354.3

### 2.7 Histology

Picro-Sirius red staining was used to visualize fibrosis as described before [8]. Briefly, samples were embedded with Tissue-Tek ® (Sakura) and sectioned in 10 μm slices. Slices were fixed with Bouin solution and subsequently stained in 0.1& (wt/vol) Sirius Red solution (Sigma-Aldrich Chemie). Sections were washed with 0.01 N HCl, Aqua dest. And dehydrated with ethanol.

Leukocytes were visualized as described before [21]. 5 μm slices were fixed with 4 % paraformaldehyde for 5 min, and washed three times with saline for 3 min. All sections were incubated with the primary antibody overnight at 4 °C. Sections were washed and reincubated with anti-Rat Cy3 secondary antibody for 1 h at room temperature followed by washing. Nuclei were stained with a dilution of 4′6′-diamidino-2-phenylindole dihydrochloride (DAPI). Cover slips were mounted using a drop of Miwiol 4–88 mounting medium. Slights were obtained with a laser confocal microscope.

### 2.8 DHE-staining

Cryosections of the ventricles were incubated with dihydroethidium (DHE, D23107, Thermo Fisher Scientific, Dreieich, Germany) dissolved in 1 *x* phosphate buffered saline for 10 min at 37 °C in a light-protected humidity chamber, then fixed with Dako Fluorescent Mounting Medium (S3023, Dako, North America Inc., USA). Slides were imaged by Keyence Microscope (BZ-X800Keyence, Neu-Isenburg, Germany). Using an excitation wavelength of 545 nm emission was recorded at 605 nm. Subsequently, digital images were analyzed using ImageJ software (Version 1.54 d).

### 2.9 Statistics

Data are expressed as indicated in the legends to the figures. Normal distribution of samples was analyzed by Shapiro-Wilk test. Normally distributed samples were subsequently analyzed by one-way ANOVA and Student-Newman-Keuls post-hoc analysis. Not normally distributed samples were analyzed by Kruskal-Wallis test and Bonferroni post-hoc analysis with correction for multiple testing or Mann-Whitney *U* test as indicated in the legends to the figures. Similarity of variance in samples from one group was analyzed by Levene test. p-Values are given in the figure legends, effects sizes, median and confidence interval in the text. Effect sizes were calculated by Cohen's D procedure and considered as strong if the Cohen's D value was above 0.8. All calculations were analyzed by SPSS 27.

## 3 Results

### 3.1 Effect of tempol and SB-3CT on l-NAME induced hypertension and hypertrophy

**Fig. 1:**
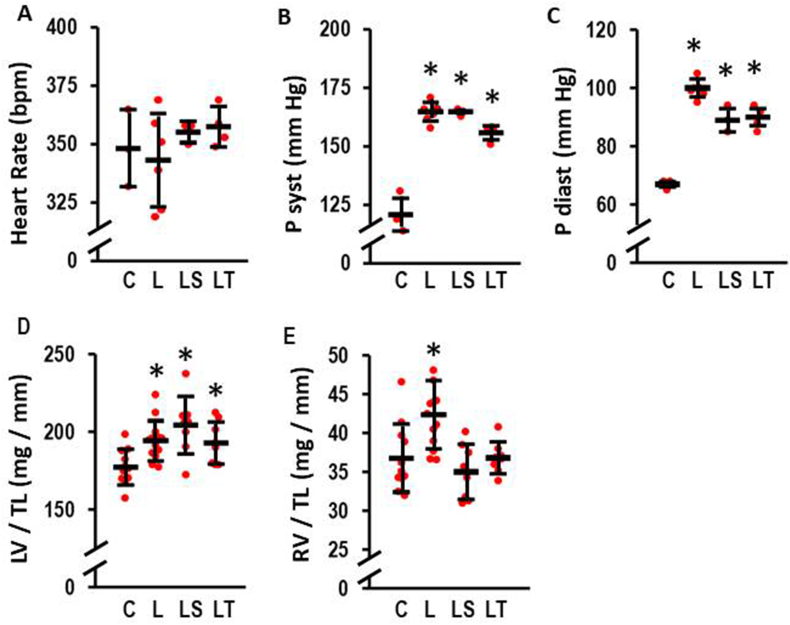
Effect of l-NAME (4 weeks; 7.5 mg/ml in tap water) on heart rate, systolic blood pressure (P syst), diastolic blood pressure (P diast), left ventricular weight (LV), and right ventricula weight (RV). Analysis was performed by tail-cuff in control rats (C, n = 3), l-NAME treated rats (L, n = 6), l-NAME treated rats with SB-3CT (LS, n = 3), and l-NAME treated rats with Tempol (LT, n = 4). Data show means ± SD in black and original data points in red. A) Comparison of heart rates in beats per minute (bpm). Kruskal-Wallis-Test: p = 0.650; B) Comparison of P syst. Kruskal-Wallis Test: p = 0.009; *, p ≤ 0.05 vs. C (exact p-values are 0.020 (L), 0.050 (LS), 0.028 (LT) with Mann-Whitney-U Test). C) Comparison of P diast. Kruskal-Wallis Test: p = 0.005; *, p ≤ 0.05 vs. C (exact p-values are 0.019 (L), 0.046 (LS), 0.032 (LT) with Mann-Whitney-U Test). Organ weights were analyzed in control (C, n = 11); l-NAME (L, n = 13); l-NAME/SB-3CT (LS, n = 8), and l-NAME/Tempol rats (LT, n = 8). D) LV weight normalized to tibia length (LV/TL); One-Way ANOVA (p = 0.002) and Student-Newman-Keuls post hoc test; *, p < 0.05 vs. C; E) RV weight normalized to tibia length (RV/TL); One-Way ANOVA (p = 0.000427) and Student-Newman-Keuls post hoc test; *, p < 0.05 vs. C. (For interpretation of the references to colour in this figure legend, the reader is referred to the Web version of this article.)

First, effects of l-NAME, Tempol, SB-3CT, and combinations thereof on blood pressure, heart rate, and heart weights were investigated. l-NAME alone had no effect on heart rates (Fig. 1A) but increased the systolic and diastolic blood pressures (Fig. 1B and C). Neither Tempol nor SB-3CT normalized blood pressure. However, mean diastolic blood pressure was slightly lower in both treatment groups compared to l-NAME (Fig. 1C).

As expected from these data, l-NAME induced an increase in LV weight normalized to tibia length (Fig. 1D). While SB-3CT slightly increased this further, Tempol did not affect the increase in LV weigth to pressure overload (Fig. 1D). Similarly, l-NAME increased RV weight normalized to tibia length (Fig. 1E). In contrast to the LV, both drugs successfully suppressed the onset of hypertrophy in the RV (Fig. 1E).

### 3.2 l-NAME induced right heart failure

**Fig. 2:**
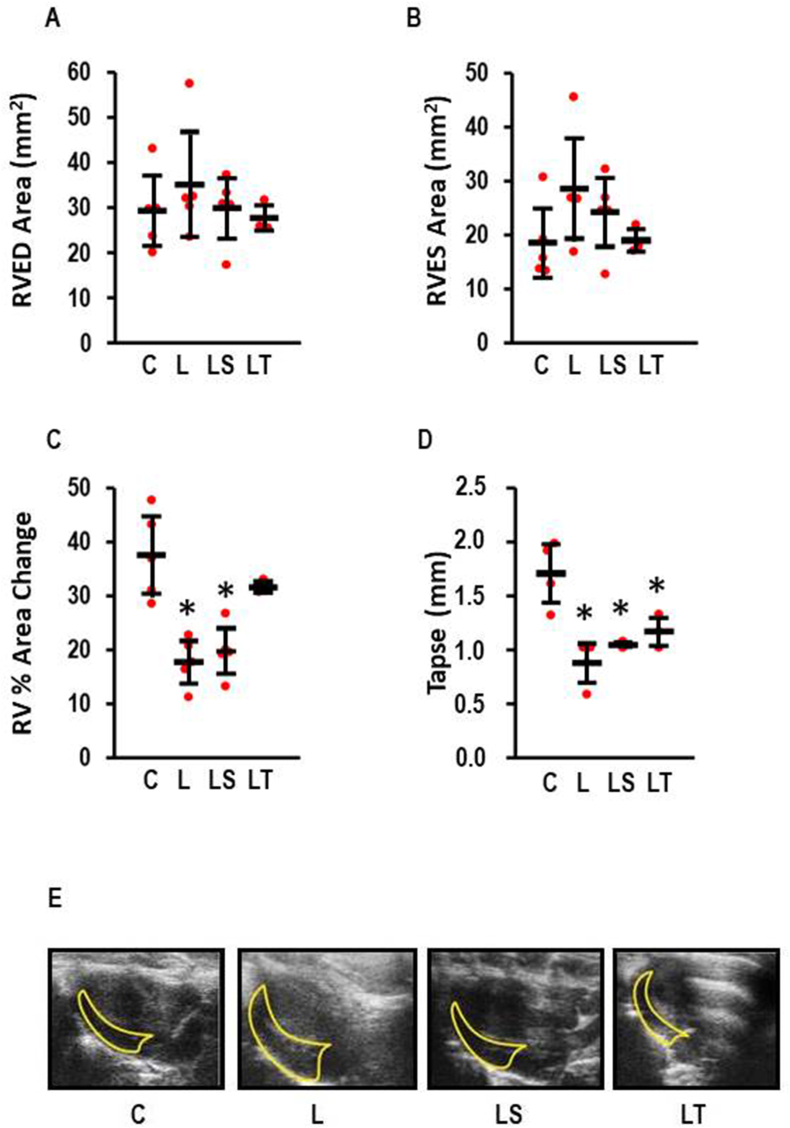
Echocardiography of the right ventricle (n = 3–6 per group). A) Right ventricular end-diastolic (RVED) area; One-Way ANOVA p = 0.669; B) Right ventricular end-systolic (RVES) area; One-Way ANOVA p = 0.212; C) Right ventricular fractional area change; One-Way ANOVA p = 0.000157; *, p < 0.05 vs. C. D) Tricuspid annular plane systolic excursion (TAPSE); One-Way ANOVA p = 0.00016; *, p < 0.05 vs. C. E) Represenative original images of a four chamber long-axis view (apical, diastolic). Data show means ± SD in black and original data points in red. (For interpretation of the references to colour in this figure legend, the reader is referred to the Web version of this article.)

RV function was analyzed by echocardiography (Fig. 2). l-NAME induced right heart failure as indicated through the reduction of right ventricular fractional area change (effect size: 3.07; CI: 1.11–4.97; p = 0.001) and the reduction of tricuspid annular plane systolic excursion (TAPSE) (effect size 3.19; 0.91–5.39; p = 0.004). In particular, Tempol improved parameters of right heart failure (Fig. 2C and D). Mean values in the SB-3CT group were also improved compared to l-NAME but to a lesser extent than with Tempol (Fig. 2C and D).

In contrast, l-NAME did not affect LV function (Table 2, Suppl. Fig. 1). The effect of either Tempol or SB-3CT on left ventricular function was not analyzed because l-NAME did not induce any functional effect on the LV.

**Table 2:** LV Function of l-NAME-treated rats (Echo Data).

	Control (n = 9)	l-NAME (n = 5)	p-Value
FS (%)	36.4 ± 7.2	35.2 ± 4.7	0.641
EF (%)	67.9 ± 7.2	62.8 ± 6.4	0.244
IVSd (mm)	0.91 ± 0.11	0.92 ± 0.11	0.901
IVSs (mm)	1.61 ± 0.48	1.66 ± 0.25	0.385
LVIDd (mm)	7.04 ± 1.60	6.98 ± 0.76	0.946
LVIDs (mm)	4.48 ± 1.00	4.50 ± 0.36	0.950
LVPWd (mm)	1.16 ± 0.14	1.09 ± 0.18	0.488
LVPWs (mm)	1.83 ± 0.49	1.88 ± 0.33	0.505

### 3.3 Effect of tempol and SB-3CT on oxidative stress

**Fig. 3:**
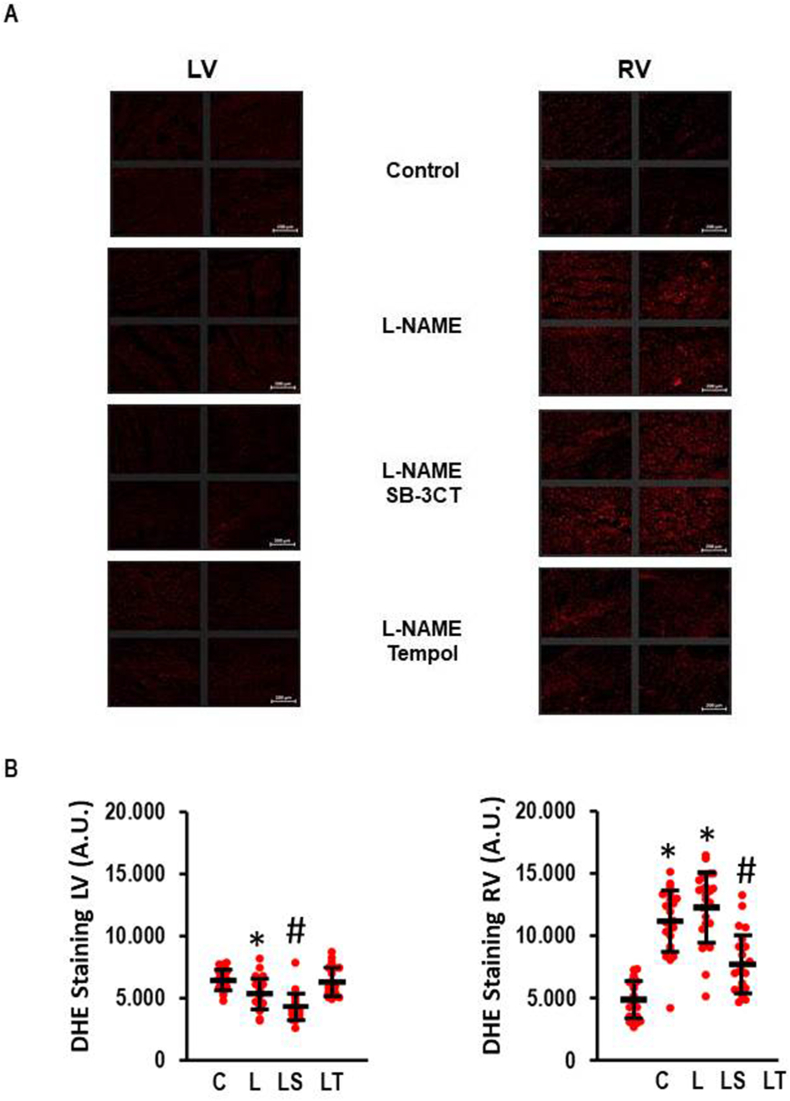
Effect of l-NAME on oxidative stress as analyzed by DHE-staining. A) Representative slides indicating oxidative stress by the appearance of red colour. Scale bars correspond to 200 μm. B) Quantiative analysis of the effect of SB-3CT and Tempol on l-NAME-induced oxidative stress. One-Way ANOVA (p < 0.000) and Student-Newman-Keuls post hoc analysis; #, p < 0.05 vs. L; *, p < 0.05 vs. C (n = 24 slices from three independent animals per group). Data show means ± SD in black and original data points in red. (For interpretation of the references to colour in this figure legend, the reader is referred to the Web version of this article.)

The difference in the l-NAME response of the RV and LV depends largely on oxidative stress that is stronger in the RV compared to the LV. Our previous finding validate this by increased DHE staining, appearance of peroxynitrite, and dimerization of tropomyosin via disulphide cross-bridges [8]. Here we used DHE staining again to analyze additionally the effect of SB-3CT on l-NAME-induced oxidative stress. Noteworthy, we confirmed our previous finding that oxidative stress occurs in the RV rather than in the LV (Fig. 3A and B). In the RV, Tempol but not SB-3CT reduced oxidative stress (Fig. 3).

### 3.4 l-NAME-induced fibrosis

**Fig. 4:**
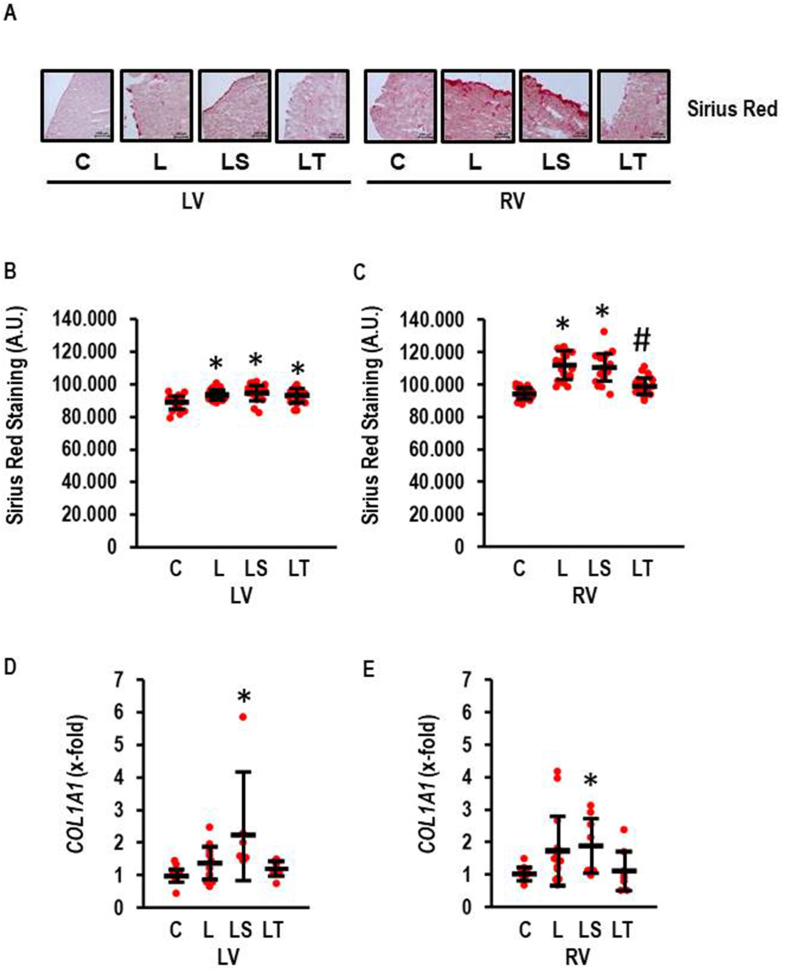
Fibrosis in l-NAME-induced RV hypertrophy. A) Sirius red staining visualizing the amount of collagen expression in both ventricle. Scale bars correspond to 100 μm. B and C) Quantification of Sirius Red Staining as arbitrary units (AU). One-Way ANOVA (p < 0.000) and Student-Newman-Keuls post hoc analysis; #, p < 0.05 vs. L, LS, C; *, p < 0.05 vs. C (n = 24 slices from four independent animals per group). D-E) *COL1A1* expression normalized to control values; LV: Kruskall-Wallis (p = 0.001); *, p < 0.05 vs. C with Bonferroni-Test with correction for multiple testing (LS vs. C: p = 0.000251); RV: Kruskal-Wallis-Test p = 0.033; *, p < 0.05 vs. C with Mann-Whitney-U-Test (LS vs. C: p = 0.010; C, n = 11, l-NAME, n = 13; LS and LT, n = 8 each). Data show means ± SD in black and original data points in red. (For interpretation of the references to colour in this figure legend, the reader is referred to the Web version of this article.)

In accordance with our previous findings [8] the RV displayed stronger fibrosis than the LV and Tempol but not SB-3CT attenuated this effect in the RV. l-NAME induced a slight effect in the LV as analyzed by Sirius Red Staining (effect size: 1.274; CI: 1.891–0.646; p < 0.000) that was not affected by SB-3CT or Tempol. l-NAME induced a strong effect in the RV on fibrosis (effect size 2.562; CI: 3.324–1.785; p < 0.000) that was attenuated by Tempol but not SB-3CT (Fig. 4A and B). The data suggest that l-NAME affects fibrosis in both ventricles in a different way. This finding was further validated by investigation of collagen mRNA expression (Fig. 4C). Here, l-NAME induced the expression of *COL1A1* and Tempol attenuated the l-NAME-induced expression of the gene whereas SB-3CT had no effect.

### 3.5 l-NAME induced MMP2 activity and modification by SB-3CT and tempol

**Fig. 5:**
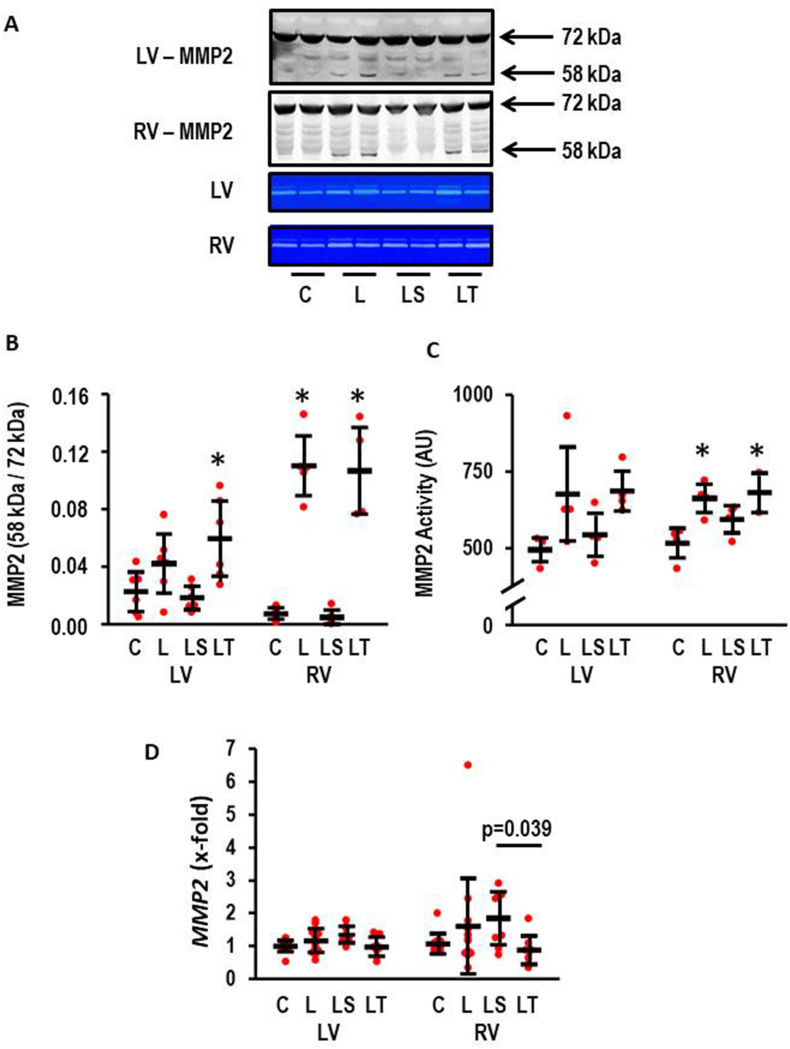
Effect of l-NAME on MMP2 activity (n = 4–6 per group). A) Representative Western Blot showing the formation of proteolytic activation by occurrence of a 58 kDa band and representative zymogram gels. B) Quantification of the Western blot; left ventricle (LV): One-way ANOVA (p = 0.008) with Student-Newman-Keuls post hoc analysis; *, p < 0.05 vs. C; right ventricle (RV): One-way ANOVA (p = 0.000000017) with Student-Newman-Keuls post hoc analysis; *, p < 0.05 vs. C; C) Quantification of zymogram gels: left ventricle: One-way ANOVA (p = 0.061); right ventricle (RV): One-way ANOVA (p = 0.017) with Student-Newman-Keuls post hoc analysis; *, p < 0.05 vs. C. D) *MMP2* expression normalized to control values; LV: One-Way ANOVA (p > 0.05) RV: Kruskal-Wallis-Test p < 0.05; with Bonferroni-Test with correction for multiple testing (LS vs. LT: p = 0.039; LS vs. C: p = 0.010; C, n = 11, l-NAME, n = 13; LS and LT, n = 8 each). Data show means ± SD in black and original data points in red. (For interpretation of the references to colour in this figure legend, the reader is referred to the Web version of this article.)

Based on the literature, we hypothesized that MMP2 activation may participate in l-NAME induced hypertrophy and failure (see Introduction). MMP2 activation occurs by proteolytic cleavage [22,23]. Therefore, we investigated whether l-NAME induced an activation of MMP2 and whether SB-3CT successfully suppresses this activation. l-NAME caused a proteolytic activation of MMP2 in the LV (effect size: 1.02; CI: 2.22 to −0.21; p = 0.107) that was successfully attenuated by SB-3CT but not by Tempol (Fig. 5A and B). Importantly, MMP2 activation was stronger in the RV (effect size: 3.15; CI: 4.89–1.35; p = 0.002). Again, SB-3CT but not Tempol attenuated proteolytic activation (Fig. 5A and B). In accordance with these findings, zymographic quantification of MMP2 activity confirmed that SB-3CT but not Tempol attenuates MMP2 activation (Fig. 5A–C). Here l-NAME increased MMP2 activity in the LV (effect size: 1.40; CI: 2.91 to −0.23; p = 0.094) and the RV (effect size: 2.67; CI: 4.64–0.60; p = 0.009). In contrast, *MMP2* expression was lowest in the l-NAME/Tempol group (Fig. 5D).

### 3.6 l-NAME induced degradation of troponin I

**Fig. 6:**
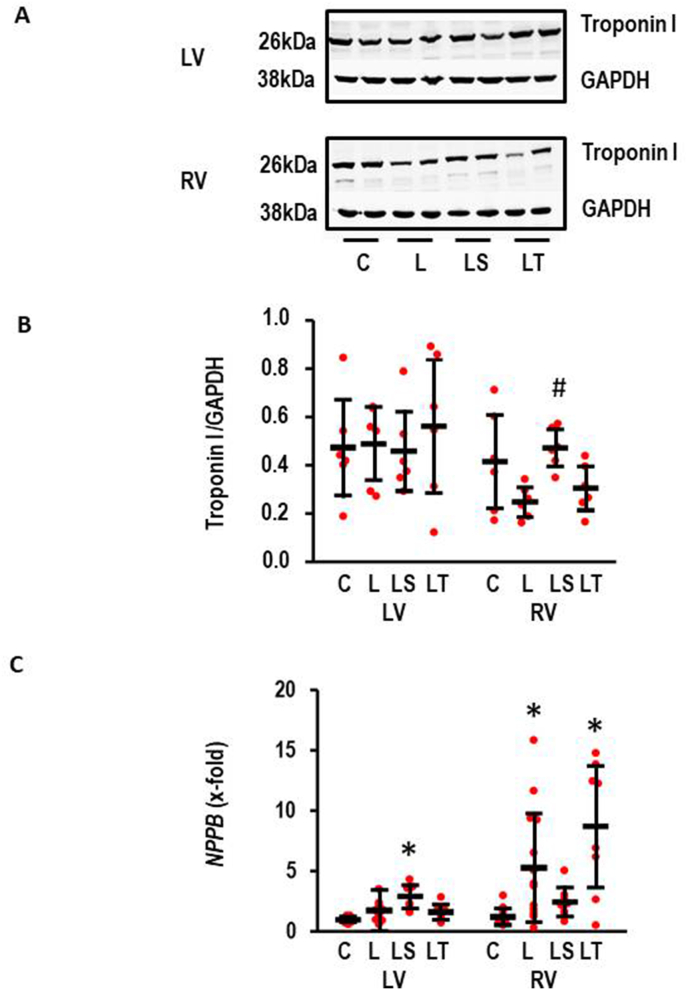
l-NAME-induced troponin degradation. A) Representative Western Blots for Troponin I and GAPDH. Samples from left ventricle (LV) and right ventricle (RV). B) Quantification of the Western Blots (n = 6 each); left ventricle (LV): One-Way ANOVA: p = 0.720; right ventricle (RV): One-Way ANOVA (p = 0.027) and Student-Newman-Keuls post hoc analysis; #, p < 0.05 vs. L. C) *NPPB* Expression normalized to control values; LV: One-Way ANOVA (p = 0.000012) and Student-Newman-Keuls post hoc test, *p < 0.05 vs. C.; RV: Kruskal-Wallis-Test p = 0.001; *, p < 0.05 vs. C with Bonferroni-Test with correction for multiple testing (L vs. C: p = 0.017; LT vs. C: p = 0.002; LS vs. C: p = 0.010; C, n = 11, l-NAME, n = 13; LS and LT, n = 8 each). Data show means ± SD in black and original data points in red. (For interpretation of the references to colour in this figure legend, the reader is referred to the Web version of this article.)

MMP2 is co-localized to troponin I and troponin I was identified previously as a target of MMP2 [4]. Therefore, we also analyzed as an indepedent read-out of MMP2 activity whether l-NAME induced a degradation of troponin I in the RV. Indeed, l-NAME reduced the expression of troponin I as quantified by the troponin to GAPDH ratio (effect size: 1.07; −0.18 – 2.27; p = 0.113). SB-3 C T but not Tempol attenuated this degradation (Fig. 6). However, l-NAME did not affect the expression of troponin I in the LV (Fig. 6).

The expression of *NPPB* was used to further characterize the phenotyp of hypertrophy (Fig. 6C). In the LV, *NPPB* expression mimicked the hypertrophic response (see Fig. 1D). l-NAME induced *NPPB* expression (effect size: 1.32; CI: 2.16–0.45; p = 0.003). Again, SB-3CT augmented the response of l-NAME whereas Tempol had no effect. In the RV, l-NAME induced the expression of *NPPB* 4.1-fold (effect size: 1.18; CI: 2.00–0.33; p = 0.006). In contrast to the LV, the l-NAME-induced expression of *NPPB* was not suppressed by SB-3CT in the RV whereas Tempol had again no effect (Fig. 6C).

### 3.7 Inflammation

**Fig. 7:**
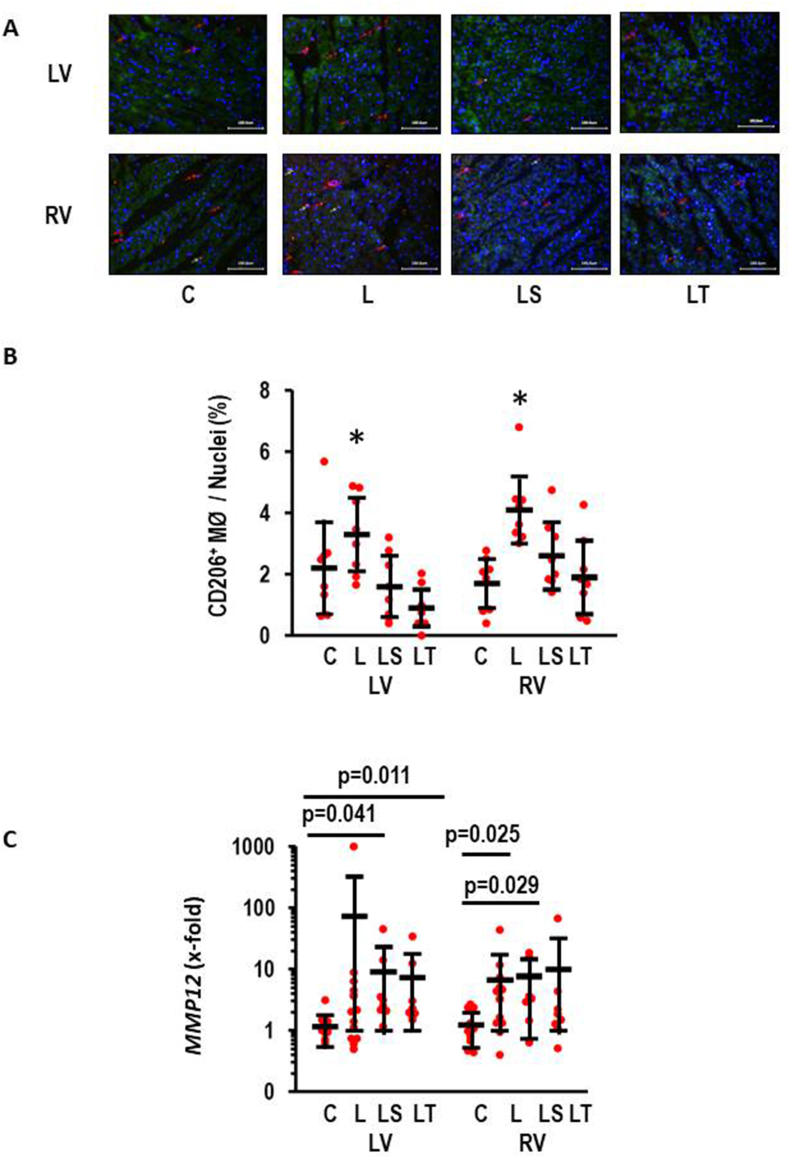
Effect of l-NAME on inflammation. A) Visualization of CD206^+^ macrophages (in red) in both ventricles. Scale bars correspond to 100 μm. B) Quantification of these visualizations (ANOVA p < 0.05 with subsequent Student-Newman-Keuls analysis for both ventricles with L different from all other gropus (*); C) *MMP12* expression in both ventricles. Kruskal-Wallis test with sunsequent Bonferonni testing for multiple testing. Exact p-values indicated. n = 8 each. Data show means ± SD in black and original data points in red. (For interpretation of the references to colour in this figure legend, the reader is referred to the Web version of this article.)

Invasion of leukocytes is another hallmark of cardiac hypertrophy. Here, we analyzed the appearance of macrophages in tissues from LV and RV (Fig. 7). l-NAME increase the quantity of CD206^+^ macrophages that was attenuated by either SB-3CT or Tempol (Fig. 7A and B). To validate these findings with an independent second method we analyzed the expression of *MMP12* in both ventricles. MMP12 is an elastase nearly exclusively expressed in macrophages and was used as a marker for inflammation [24]. l-NAME induced a strong increase in *MMP12* expression in both ventricles. In the LV *MMP12* expression was induced 71.0-fold (effect size 0.37; 1.15 to −0.41; p = 0.354) and in the RV l-NAME induced *MMP12* expression 5.3-fold (effect size: 0.66; CI: 1.45–0.14; p = 0.105). In both ventricles there was a large inter-individual variability. However, although both drugs reduced *MMP12* expression in the LV they did not affect the l-NAME effect in the RV (Fig. 7C).

### 3.8 l-NAME and uncoupling protein (UCP)2 in RV

**Fig. 8:**
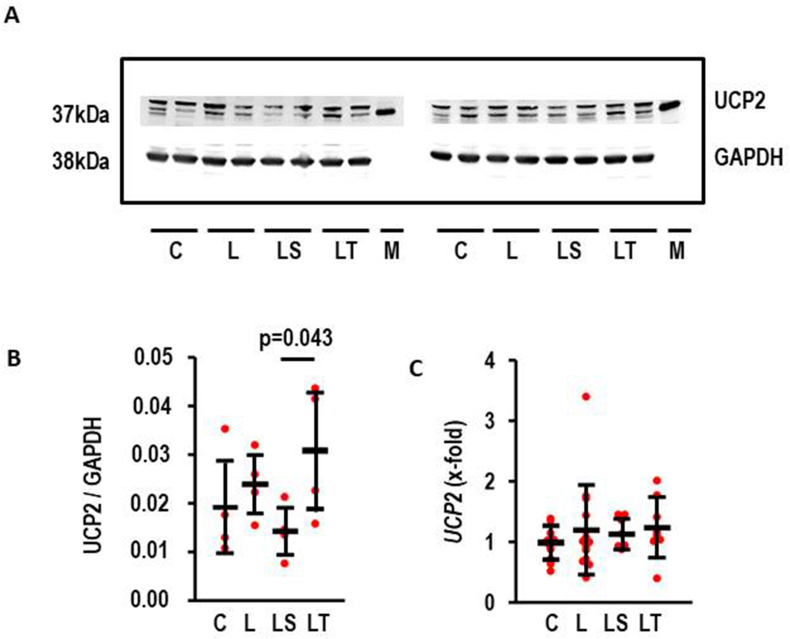
Effect of L-NAMEon UCP2 expression in RV A) Representative Western Blot for UCP2 and GAPDH. B) Quantification of the Western blot (each group: n = 4); LS vs. LT: p = 0.043 (Mann-Whitney-U Test. C) *UCP2* expression in the RV; ANOVA p > 0.05. Data show means ± SD in black and original data points in red. (For interpretation of the references to colour in this figure legend, the reader is referred to the Web version of this article.)

**Fig. 9:**
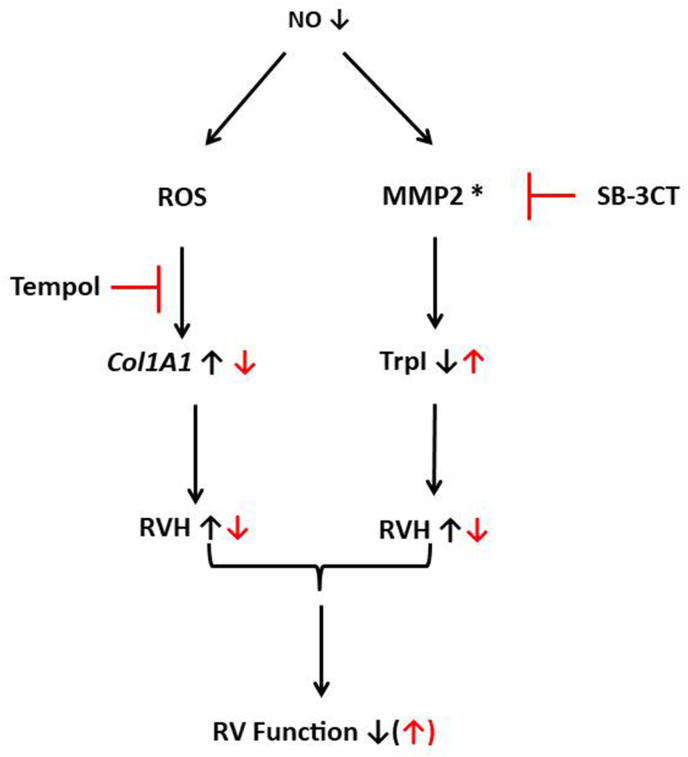
Summary of our results explaining the different targets of inhibition of MMP2 by SB-3CT or activation of SOD by Tempol. NO-deficiency (in black) increases the amount of reactive oxygen species (ROS) and activates MMP2 (as indicated by an asterisk). Inhibition of oxidative stress by Tempol (in red) attenuates l-NAME-induced collagen expression whereas inhibition of MMP2 activity (in red) attenuates troponin degradation. l-NAME-induced collagen expression and troponin degradation lead to RV hypertrophy (RVH) that can be attenuated by either Tempol or SB-3CT. Consequently, inhibition of only one of the identified pathways is insufficient to ameliorate RHF completely. (For interpretation of the references to colour in this figure legend, the reader is referred to the Web version of this article.)

UCP2 is the main UCP isoform in the heart linking metabolism to oxidative stress [25]. UCP2 protein expression was slightly induced in the l-NAME-treated group (Fig. 9; effect size: 0.51; CI: 1.91 to −0.92; p = 0.495) but more importantly stronger expressed in l-NAME/Tempol vs. l-NAME/SB-3CT (Fig. 8B). In contrast, l-NAME did not modify *UCP2* expression (Fig. 8C). The data suggest that oxidative stress destabilizes UCP2 protein expression.

## 4 Discussion

Our study was aimed at identifying the role of MMP2 in l-NAME-induced right heart failure. l-NAME induced increased systemic blood pressure in these rats and this caused compensatory LV hypertrophy. l-NAME also induced RV hypertrophy but here in a dysfunctional way. We showed before, that the left ventricle upregulates its oxidative defense by induction of SOD2 whereas the right ventricle cannot adapt to oxidative stress [8]. Therefore, we administered a SOD-mimetic and expected that this will ameliorate right heart failure in this model. Furthermore, we hypothesized that oxidative stress will affect fibrosis via activation of MMP2. Therefore, we administered also a MMP2 inhibitor in the same model. Our main new findings from this study are as follows: Reduction of oxidative stress via Tempol effectively reduced RV hypertrophy, right heart fibrosis (shown histologically by reduced Sirius Red Staining and by reduced expression of *COL1A1*), reduced the abundance of CD206^+^ macrophages in the RV, and increased the abundance of UCP2 in the RV. However, improving the oxidative stress defense via the SOD mimetic did not reduce the expression of the macrophage-specific elastase MMP12, did not attenuate the activation of MMP2 and the consequences of MMP2 activation such as degradation of troponin I. In summary, improving the oxidative defense in the right ventricle by administration of a SOD mimetic improved some but not all consequences of NO-deficiency in this right heart failure model. These improvements were directed against pro-fibrotic signal pathways. The second important new finding of our study is as follows: We show that oxidative stress is not involved in MMP2 activation in right heart failure. Furthermore, we show that MMP2 targets are mainly linked to cardiomyocytes. MMP2 inhibition attenuates RV hypertrophy and *NPPB* expression in the RV and decreased troponin I degradation. MMP2 inhibition did not affect any of the fibrosis or inflammatory markers. In summary, inhibiting MMP2 activity rescued some but not all of the l-NAME-induced mal-adaptations in the RV. Overall, our results suggest that NO deficiency in the RV induces pathways linked to oxidative stress and fibrosis as well as those linked to MMP2. Moreover, MMP2 seems to be predominantly acting on cardiomyocytes whereas oxidative stress predominantly affects fibrosis. This is important to know because independently both pathways have strong effects on individual molecules but did not strongly mitigate right ventricular failure when inhibited separately. Nevertheless, the combined inhibition of oxidative stress and MMP2 activation should be an attractive strategy for future studies. Figure 9 summarizes our findings.

Irrespectively of the l-NAME induced effect on the RV, the effect on the LV is less severe. l-NAME induced severe hypertension and this subsequently triggered myocardial hypertrophy. Of note, neither SB-3CT nor Tempol reduced the increase in blood pressure. We previously showed that l-NAME induces hypertension within two weeks with only moderate further increase in weeks three and four [8]. The slightly lower blood pressure in our treatment groups may suggest that this late but additional increase in blood pressure was attenuated without normalizing blood pressure. In our recently published in vitro study based on adult terminal differentiated cardiomyocytes from rat ventricles we were able to show that MMP2 suppresses hypertrophic growth [16]. In line with this, inhibition of l-NAME-dependent MMP2 activation further increased left ventricular hypertrophy as quantified by higher LV weight and *NPPB* expression in the LV. Our former study used ventricular cell preparations. Such preparations contain mainly left ventricular myocytes because the left ventricular mass (here: LVW/TL: 177 mg/mm; Fig. 1D) exceeds that of the free right ventricle (here: RVW/TL: 37 mg/mm; Fig. 1E). Therefore, this finding supports our previous in vitro finding in vivo. In the context of our study reported here, it is important to notice that a similar effect was not seen in the RV. Obviously, inhibition of MMP2 can reduce RV weight but increases that of the LV. Again the different responsiveness to MMP2 between both ventricles adds another puzzle that explains the different response of the ventricles to pressure load. Collectively, these results underline the need of viewing the RV myocardium as an independent entity which must be taken into account for the development of new therapeutic strategies.

Heart failure depends at least in part on the recruitment of inflammatory cells. MMP12 is a macrophage-specific elastase [22,23]. As an elastase it cleaves elastin and thereby pro-fibrotic cytokines such as TGF-β_1_ are released which contribute to pro-fibrotic remodeling of the organ. In the l-NAME model used here, ventricular expression of *MMP12* is strongly induced as validated by RT-PCR. This might reflect macrophage invasion or differentiation. Here we found an effect of Tempol and MMP2-inhibition on the number of CD206^+^ macrophages, but the important expression of MMP12 displayed another picture. Interestingly, in the LV Tempol and SB-3CT reduced MMP12 expression. In the LV, hypertrophy was still in a compensatory state. However, in the RV, developing maladaptive remodeling, neither SB-3CT nor Tempol reduced *MMP12* expression. Although we do not know mechanistically how both drugs lower *MMP12* expression in the LV this is, again, a remarkable difference in the stress response of both ventricles. Finally it must be noted that large inter-individual variability exists in *MMP12* induction. In order to understand the role of inflammation in LV and RV hypertrophy, different types of macrophages must be stained in future studies as MMP12^+^ cells and CD206^+^ cells may overlap but not be identical cell populations. Nevertheless, we do not have evidence right now that immune cells contribute to the ventricle-specific differences. This conclusion is also validated in another rat model that compared mal-adaptive hypertrophy between right and left ventricles in which increased fibrotic marker expression but no induction of inflammatory pathways were found [3].

MMP2 activation by ROS is located at least in part at the site of mitochondria [9]. Mitochondria are mechanistically important for cardiac pathology [25]. We have recently identified downregulation of the mitochondrial uncoupling protein 2 (UCP2) in adaptive hypertrophy. However, super-induction of UCP2 occurs at the time of transition to heart failure [26]. Cardiomyocytes express two different isoforms of UCPs, namely UCP2 and UCP3. Both isoforms have an extremely low half-life suggesting mRNA expression corresponds to protein values [27]. l-NAME did not affect the expression of *UCP2*.

In contrast to UCP2 mRNA, the protein expression of UCP2 was affected by l-NAME/Tempol in the RV. This suggests that oxidative stress determines UCP2 half-life. As a consequence of this, UCP2 protein expression is highest in the l-NAME/Tempol group but lowest in the l-NAME/SB-3CT group. Based on our previous studies on UCP2 in pressure overload this is contradictory to our findings, as banding of the pulmonary artery (PAB) was less detrimental in UCP2 knockout mice [28]. However, the cell-type specific expression of UCP2 differs remarkable between rats and mice, making a transfer of the conclusions from the mouse to the rat model rather speculative. Future studies with UCP2 knockout rats will clarify this point. As it stands the new finding in this part of our study is the identification of a ROS-dependent effect on UCP2 protein degradation.

In principle one may consider the use of female rats in this project as a study limitation. The decision to use only female rats in this study is in agreement with the local authorities and aimed at to reduce sex-dependent increases in variability. Using rats with different sex will increase the numbers of rats. The protocol was recently be used in our previous publication as well (Ref. 8). However, we also performed a PAB banding study vs. AOB (Ref. 3, Manuscript attached) in which we used male and female rats. These studies did not show any sex-dependent differences but comparable pathways (namely fibrosis). Therefore, we do not expect any impact of sex on these results.

In summary, this study adds new information how differences in oxidative defense adaptation to pressure overload between RV and LV lead to less stress tolerance of the RV. Considering the ability to increase the abundance of muscluar mass to adapt to pressure overload (compensatory hypertrophy) it is noteworthy that both ventricles respond differentially to oxidative stress and MMP2 activity with respect to organ growth. Furthermore, the inability of the RV to improve its oxidative defense system under conditions of pressure load activates strongly the ROS-fibrosis axis that affects geometry and biophysics of the RV. The strong activation of MMP2 in the RV targets the sarcomer structure affecting contractile function as key proteins such as troponin I are reduced. Furthermore, neither MMP2 nor oxidative stress contribute to the induction of MMP12 in the RV whereas a large part of MMP12 activity in the left ventricle are sensitive to these stressors.

## Funding

This research was funded by Deutsche Forschungsgemeinschaft (DFG, German Research Foundation), grant number 268555672 – SFB 1213, project B05. The Ministry for Innovation and Technology in Hungary provided funding to this study (2020–1.1.5-GYORSITOSAV-2021-00011) and was also supported by RRF-2.3.1-2022-00003 “National Heart Laboratory, Hungary. Further funding came from the Hungarian National Scientific Research Fund (OTKA-138223). T.S. was supported by the Cooperative Doctoral Programme (KDP-2020) of the Ministry fir Innovation. P.B. was supported by the Janos Bolyai Research Scholarship of the Hungarian Academy of Science (bo_481_21), and by the New National Excellence Program of the Ministry of Human Capacities (UNKP-23-5-SZTE-704).

## CRediT authorship contribution statement

**Rolf Schreckenberg:** Writing – review & editing, Validation, Methodology, Investigation, Formal analysis, Data curation, Conceptualization. **Rainer Schulz:** Writing – review & editing, Funding acquisition, Conceptualization. **Nadja Itani:** Investigation. **Peter Ferdinandy:** Methodology, Conceptualization. **Peter Bencsik:** Validation, Data curation. **Tamara Szabados:** Investigation. **Susanne Rohrbach:** Formal analysis, Data curation. **Bernd Niemann:** Investigation. **Klaus-Dieter Schlüter:** Writing – review & editing, Writing – original draft, Validation, Supervision, Resources, Funding acquisition, Data curation, Conceptualization.

## Declaration of competing interest

None.

## Data Availability

Data will be made available on request.
