# Supplementary figures and images for: Inhibition of MMP2 activity mitigates N-omega-nitro-l-arginine-methyl ester (l-NAME)-induced right heart failure
Source: Redox Biol. 2024 Aug 15;76:103308. doi: 10.1016/j.redox.2024.103308 (PMC11381879; doi:10.1016/j.redox.2024.103308)

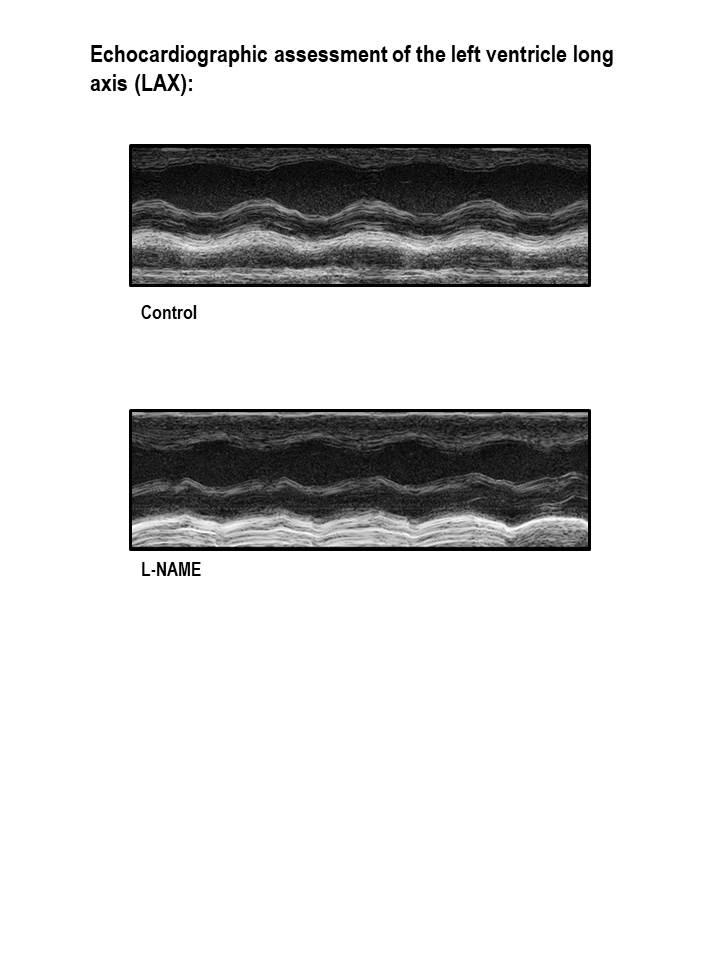


Supplement Fig. 1

Supplement: Multimedia component 1 [file mmc1.docx]
